# Genomics and the Prevention and Control of Common Chronic Diseases: Emerging Priorities for Public Health Action

**Published:** 2005-03-15

**Authors:** Muin J Khoury, George A Mensah

**Affiliations:** Office of Genomics and Disease Prevention, Coordinating Center for Health Promotion, Centers for Disease Control and Prevention; National Center for Chronic Disease Prevention and Health Promotion, Coordinating Center for Health Promotion, Centers for Disease Control and Prevention, Atlanta, Ga

The completion of the Human Genome Project in 2003 continues to raise expectations on near-term applications of human genome discoveries in personalized disease prevention, especially in the area of common chronic diseases (1,2). In fact, almost daily we are confronted with stories of scientific discoveries of human genetic variants that are suggested to affect our risks for one or more of the major common chronic diseases. (See Table 1 for an illustrative sample of news stories published online during December 2004 [3].) Yet the immediate significance of most of these discoveries remains elusive. Despite the scientific excitement and the predictions for personalized prevention and drug treatment, the promise of human gene discovery for health promotion and disease prevention is yet to be fulfilled (4).

Increasingly, public health practitioners from academic, government, and other organizations have taken a proactive leadership role in assessing the relevance of this technology to population health and to community-based interventions (a new field often referred to as public health genetics, or genomics) (5). This issue of *Preventing Chronic Disease* contains several articles illustrating various processes developed and applied by schools of public health and state health departments to evaluate the role of genomics and its relevance to the prevention of chronic diseases in the population (6-11).

Johnson et al (6) demonstrate the feasibility and success of using family history as a simple genomic tool to inform and motivate high-risk families to make long-term lifestyle behavior changes for preventing a variety of chronic diseases. Annis et al (7) show that existing population-based databases contain valuable genomic information and can serve as a reliable source for chronic disease program recommendations for early detection, prevention, and risk assessment. Irwin et al (8) examine the genomic content of state Comprehensive Cancer Control programs and show that many states have genomic components in their written plans. Importantly, about 67% of programs that included family history in their plans have already begun implementing their stated goals. Harrison et al (9) describe a process for synthesizing genomics information and for sharing knowledge and lessons learned. Novel educational approaches, such as the one presented by Theisen et al (10), and innovative training tools, such as those highlighted by Bodzin et al (11), will be crucial in efforts to provide continuing education for the public and health care professionals. A central theme in all of these papers is the importance of family history as a tool for chronic disease prevention and health promotion.

## Why should public health focus on family history in the genomics era?

In November 2004, the United States Surgeon General launched a public education campaign urging all citizens to know their family medical history and to discuss it with health care providers using an online family history collection tool (12). With all the excitement about genomics, one may wonder why we are still using an old-fashioned tool such as family history (13). Nonetheless, several basic facts about family history make it ideal for use in public health practice:

1) A history of one or more chronic diseases is common in the population, although awareness of the details and accurate reporting of the disease may be suboptimal.

2) Family history is the most consistent risk factor for almost all human diseases across the lifespan (14). Thus, the presence of a disease in a family, especially among first-degree relatives, increases the risk for that disease.

3) Family history reflects the complex interaction among many shared genes, shared behaviors, shared cultures, and shared environments among families, all of which could also be disease risk factors. In fact, only a small fraction of people with family history of a common chronic disease have a "genetic disease" (with high lifetime risk of disease). A total of 188 such diseases have been identified as of 2004, accounting for a relatively small burden of chronic disease (15). Most people with family history of a disease have a moderately increased risk of the disease. 

4) Although family history cannot be changed, knowledge of it provides an opportunity to personalize and target our myriad disease prevention and health promotion messages (12,16).

5) Today, family history is the best genomic tool available, and compared with other genetic tests, it can be relatively inexpensively collected.

Although eliciting family history should be a routine component of patient medical records and encounters with health care providers, the completeness of such information and its use in practice are less than optimal. Despite the implications of family history for public health, the public's knowledge, attitudes, and behaviors related to family history have some way to go. In a recent national survey of 4345 adults, the Centers for Disease Control and Prevention (CDC) found that although most respondents (97%) considered knowledge of family history either very important or somewhat important to their personal health, a strikingly smaller proportion (30%) reported actively engaging in collection of information to develop a family health history (17). Routine collection and use of the family history by clinicians remains suboptimal. Acheson et al have shown that family history is discussed in only half of new visits to primary care physicians and 22% of follow-up visits. Also, the average duration of the family history discussion is only 2.5 minutes, focusing mostly on psychosocial and not health-related issues (18). More recently, Walter et al reviewed lay understanding of familial risk for common chronic diseases and how each person's sense of disease vulnerability depends not only on family history but also on personal models of disease causation and inheritance (19). These findings underscore the need for more public and health provider education to improve the effectiveness of using family history as a risk communication tool for personal health and disease prevention.

Despite the importance of family history for health promotion, our public health strategy in preventing common chronic diseases continues to be a one-size-fits-all approach to promoting healthy lifestyles in the population at large. Most people do not get enough physical activity, are overweight, and do not adhere to health screening recommendations. With development and validation of the right family history tools, public health can begin to develop, test, and apply personalized prevention messages. Because a large fraction of the population has a family history of one or more common diseases, augmenting and not replacing the population approach to prevention with an approach focused on higher-risk families may help achieve overall population health goals. For example, we know from population studies conducted in Utah that 14% of families have almost 72% of the burden of early heart disease (under 50 years of age) and 48% of the burden of all heart disease in the whole population (20). A distinct advantage of a family-centered approach to prevention is that it does not focus exclusively on genetic factors but works within an overall framework of biologic and sociocultural relationships to effect behavioral change and risk factor reduction. As discussed in this issue by Johnson et al (6), family history can begin to build a bridge between the one-size-fits-all approach to prevention and the one-person-at-a-time approach to genetics.

## What are the emerging public health priorities in genomics?

Three emerging priorities for public health action in genomics highlighted in this issue of *Preventing Chronic Disease* are 1) the conduct and support of population-based research and databases in genomics and health; 2) the development of the evidence base for genomic applications in health promotion and disease prevention; and 3) the assurance of an adequate public health capacity in genomics. These public health genomics priorities provide a road map for long-term translation of human genome discoveries into chronic disease prevention and health promotion across the lifespan.

**1) Conduct and support of population-based research and databases on genomics and health.** Generally, additional public health research is needed to asses the impact of the thousands of genetic variants (and their interactions with modifiable risk factors) on the burden of chronic diseases (incidence and prevalence as well as morbidity, disability, and mortality). Although gene–disease associations continue to be investigated in the context of family studies, association studies using mostly case-control designs are becoming more common. As shown in Table 2 for a sample of common chronic diseases, the number of published epidemiologic articles on gene–disease association is increasing over time. These data are derived from the Genomics and Disease Prevention Information System (GDPInfo) (21), an online searchable and continuously updated information system developed by the CDC. Between October 1, 2000, and December 30, 2004, GDPInfo captured records of 13,858 published epidemiologic articles on genes and disease outcomes. The population-level implications of findings from many such studies are unclear. Often, the potential importance of a reported association is impossible to evaluate because basic population-based genotype prevalence data are not available. The CDC and many collaborators in the Human Genome Epidemiology Network (HuGE Net) are currently developing methodologic guidance and systematic reviews for integrating data from these studies and developing inference for research, policy, and practice (22).

Another example of the use of national surveys is the existing DNA repository containing specimens from more than 7000 participants in the second phase of National Health and Nutrition Examination Survey III (1991–1994). The CDC determined the prevalence of gene variants associated with hereditary hemochromatosis (23,24). The CDC in collaboration with the Juvenile Diabetes Research Foundation and others created the type 1 diabetes DNA repository to study genetic risk factors for sequelae of type 1 diabetes (25-27). Also, the CDC developed a DNA repository as part of the population-based National Birth Defects Prevention Survey (28).

In addition to national surveys, state public health programs can provide valuable population data to assess the impact of genes on the burden of chronic disease. An example of state-based data collection for chronic disease is the cancer registries that are a component of state-based comprehensive control programs (see Irwin et al in this issue). These registries can truly provide a population-level assessment of the impact of family history and individual genes on the burden of cancer in the United States.

**2) Development of the evidence base for genomic applications in health promotion and disease prevention.** Public health is beginning to evaluate the added value of using genetic tests as tools for disease prevention. For decades, public health practice has downplayed the "high risk" model of prevention in favor of a population approach, which may not benefit most individuals but can have a large impact on the burden of disease (29,30). For example, a small downward shift in mean serum cholesterol distribution could reduce the burden of coronary heart disease in the population more than treating people with high cholesterol levels, since most of the burden of heart disease occurs among persons with values in the normal range (29). As the discussion of family history illustrates, we can combine a high risk strategy with a population strategy to achieve overall public health goals. Nevertheless, the use of individual genes as risk factors for disease or the use of a combination of genetic variants along biologic pathways (so-called genomic profile) for testing for chronic disease susceptibility should be quantitatively evaluated for its potential impact on individuals and the population (31,32). In general, existing quantitative evaluations of combinations of genetic variants and their expressions are still in the theoretical realm and are based on many assumptions that have not been validated.

As in other areas of health practice, public health is becoming the convener of multidisciplinary deliberations needed to determine the role, if any, of genomics information, above and beyond a population approach to the prevention of chronic diseases (e.g., smoking control programs, diet, physical activity, early detection programs). The recent surge of direct-to-consumer marketing of genetic tests, such as genomic profiles for susceptibility to cardiovascular disease and bone health (33) and for testing for breast and ovarian cancer (34), will increasingly necessitate a public health response for building the evidence base for use of genomic applications in population health and for measuring the impact of test marketing on consumers' and providers' knowledge, attitudes, and behaviors.

For the past five years, the CDC and many collaborators have developed model approaches for obtaining and synthesizing available information on genetic tests. The Foundation for Blood Research developed key data elements needed for genetic tests by intended use and applied a model approach to five conditions (35). The CDC and many partners are currently exploring the development of a more sustainable public–private partnership process to summarize evidence and identify gaps in knowledge to stimulate further research. In addition, public health assessment will be needed to monitor current and future levels of use of genetic tests, as well as knowledge, attitudes, and behaviors of consumers and health care providers. A population-based approach in collecting valid clinical and laboratory data will ensure that consumers, practitioners, and policy makers have access to timely and current information on genetic tests and their impact on the public's health. These efforts will also allow a smoother integration of validated genetic tests into practice.

One example of a public health assessment in genomics is a 1997 expert panel workshop jointly held by the National Institutes of Health (NIH) and the CDC to explore issues on population screening for iron overload due to hereditary hemochromatosis (a small but preventable cause of multiple chronic diseases including heart disease, liver cancer, diabetes, and arthritis) (36). This collaboration led to the identification of important gaps in research, funding of further studies, and implementation of a nationwide physician training program that promotes early detection of hemochromatosis (37).

**3) Assurance of an adequate public health capacity in genomics.** Clearly, the integration of genomic information into practice and programs requires resources, a competent workforce, a robust public health system that can address health disparities, and an informed public. Educational and planning resources for public health genomics have been developed over the past three years (11). The Association of State and Territorial Health Officials' guide to genomics for public health practitioners (38) and the Web-based introduction to genomics (*Six Weeks to Genomic Awareness*) developed by the University of Michigan (39) are examples of the available resources. Another useful state policy guide for genomics and chronic diseases was developed by the Partnership for Prevention™, a public–private coalition focused on disease prevention and health promotion (40).

A 2003 report by the Institute of Medicine identified genomics as one of the eight crosscutting priorities for the training of all public health professionals in the 21^st^ century (41). In October 2003, the Association of Schools of Public Health administered an online survey to representatives of all 33 accredited U.S. schools of public health. The survey provided a baseline assessment of the extent to which the schools were offering curriculum content in the eight areas recommended by the Institute of Medicine, including genomics (42). Although 52% of the schools offer courses in genomics, only 15% of the schools require genomics to be part of their core curriculum, clearly the lowest figure for all eight crosscutting areas (highest was policy with 79%), indicating a definite need for improved integration of genomics into public health education (42).

Over the past five years, the CDC has promoted the integration of genomics across all public health functions, including training and workforce development. In collaboration with many partners, the CDC hosted the development of public health workforce competencies in genomics (43), established three Centers for Genomics and Public Health at schools of public health to develop training (44), provided funding and technical assistance to state and local health departments (45), and actively engaged in offering training and career development opportunities in genomics and public health. As discussed by Dr Bill Roper, previous Dean of the School of Public Health at the University of North Carolina at Chapel Hill, at the CDC public health genomics symposium in May 2003, genomics is becoming an integral component of the concept of "public health preparedness" in the 21^st^ century (46).

## Conclusion

Although a nascent field, the application of genomics to chronic disease prevention and health promotion is already occurring at the state and local levels. Success in this endeavor will require building appropriate capacity and maintaining a skilled public health workforce competent in the evaluation and use of genomic information for preventing common chronic diseases. Of equal importance is the development of innovative tools and evidence-based processes that allow the differentiation between genomic technology that is ready for use in population health and technology that is not ready for prime time. Health policies need to be developed to ensure the appropriate use of genomic information for health promotion while avoiding psychosocial and financial harms to individuals and population groups. As exemplified in this issue of *Preventing Chronic Disease*, we believe that the continued leadership and collaboration among schools of public health, state health departments, and other public health partners will take us a long way to ensure that human genomic information will be used for preventing chronic diseases and promoting health in individuals, families, and communities.

## Figures and Tables

**Table 1:** Examples of News Stories on Human Genome Discoveries Relevant to Common Chronic Diseases^a^

**Story Headline**	**Source**	**Date**
Faulty gene signaling linked to Crohn's	*HealthDay News*	Dec 28, 2004
Genetic difference at opiate receptor gene affects a person's response to alcohol	*Medical News Today*	Dec 15, 2004
Important discovery of gene involved in breast and prostate cancer	*PR Newswire*	Dec 15, 2004
Mutant gene linked to treatment-resistant depression	NIH news release	Dec 14, 2004
Molecular test can predict both the risk of breast cancer recurrence and who will benefit from chemotherapy	NCI news release	Dec 10, 2004
Important genetic risk factor for amyotrophic lateral sclerosis	*News-Medical.Net*	Dec 6, 2004
Association of vitamin D receptor gene polymorphisms with childhood and adult asthma	*RedNova News*	Dec 2, 2004

a Web-based stories posted on the CDC Genomics and Health Weekly Update, December 2004. Available from http://www.cdc.gov/genomics/update/current.htm. NIH indicates National Institutes of Health; NCI indicates National Cancer Institute.

**Table 2:** Number of Gene–Disease Association Studies Reported in the Medical Literature, by Year and Disease, CDC Genomics and Disease Prevention Information System, 2001–2004^a^

**Disease**	**No. published articles**			

	**2001**	**2002**	**2003**	**2004**
Coronary heart disease (and stroke)	197	205	262	238
Diabetes	128	154	156	165
Breast cancer	61	111	136	146
Colorectal cancer	53	55	46	70
Lung cancer	35	52	60	55
Alzheimer’s	88	100	111	145
Asthma	38	40	49	71

a Search was conducted on December 30, 2004, at http://www2a.cdc.gov/genomics/GDPQueryTool/frmQueryAdvPage.asp.

## References

[1] Guttmacher AE, Collins FS (2003). Welcome to the genomic era. N Engl J Med.

[2] Bell J (2004). Predicting disease using genomics. Nature.

[3] Centers for Disease Control and Prevention Genomics & health weekly update.

[4] Haga S, Khoury MJ, Burke W (2003). Genomic profiling to promote a healthy lifestyle: not ready for prime time. Nat Genet.

[5] Khoury MJ, Burke W, Thomson E (2000). Genetics and public health in the 21st century: using genetic information to improve health and prevent disease.

[6] Johnson J, Giles RT, Larsen L, Ware J, Adams T, Hunt SC (2005). Utah's Family High Risk Program: bridging the gap between genomics and public health. Prev Chronic Dis [serial online].

[7] Annis AM, Caulder MS, Cook ML, Duquette D (2005). Associations among diabetes, family history, and other demographic and risk factors among participants of the National Health and Nutrition Examination Survey 1999–2002. Prev Chronic Dis [serial online].

[8] Irwin DE, Zuiker ES, Rakhra-Burris T, Millikan RC (2005). Review of state Comprehensive Cancer Control plans for genomics content.. Prev Chronic Dis [serial online].

[9] Harrison TA, Burke W, Edwards KL (2005). The asthma consultative process: a collaborative approach to integrating genomics into public health practice. Prev Chronic Dis [serial online].

[10] Theisen V, Duquette D, Kardia S, Wang C, Beene-Harris R, Bach J (2005). Blood pressure Sunday: introducing genomics to the community through family history. Prev Chronic Dis [serial online].

[11] Bodzin J, Kardia SLR, Goldenberg A, Raup SF, Bach JV, Citrin T (2005). Genomics and public health: development of Web-based training tools for increasing genomic awareness. Prev Chronic Dis [serial online].

[12] U.S. Department of Health and Human Services U.S. Surgeon General's family history initiative.

[13] Guttmacher AE, Collins FS, Carmona RH (2004). The family history--more important than ever. N Engl J Med.

[14] Yoon PW, Scheuner MT, Khoury MJ (2003). Research priorities for evaluating family history in the prevention of common chronic diseases. Am J Prev Med.

[15] Scheuner MT, Yoon PW, Khoury MJ (2004). Contribution of Mendelian disorders to common chronic disease: opportunities for recognition, intervention, and prevention. Am J Med Genet C Semin Med Genet.

[16] Khoury MJ (2003). Genetics and genomics in practice: the continuum from genetic disease to genetic information in health and disease. Genet Med.

[17] Yoon PW,  Scheuner MT, Gwinn M, Khoury MJ, Jorgensen C, Hariri S (2004). Awareness of family health history as a risk factor for disease --- United States, 2004. MMWR.

[18] Acheson LS, Wiesner GL, Zyzanski SJ, Goodwin MA, Stange KC (2000). Family history-taking in community family practice: implications for genetic screening. Genet Med.

[19] Walter FM, Emery J, Braithwaite D, Marteau TM (2004). Lay understanding of familial risk of common chronic diseases: a systematic review and synthesis of qualitative research. Ann Fam Med.

[20] Hunt SC, Gwinn M, Adams TD (2003). Family history assessment: strategies for prevention of cardiovascular disease. Am J Prev Med.

[21] Centers for Disease Control and Prevention Genomics and disease prevention information system (GDPInfo).

[22] Centers for Disease Control and Prevention The Human Genome Epidemiology Network (HuGENet).

[23] Cogswell ME, Gallagher ML, Steinberg KK, Caudill SP, Looker AC, Bowman BA (2003). HFE genotype and transferrin saturation in the United States. Genet Med.

[24] Steinberg KK, Cogswell ME, Chang JC, Caudill SP, McQuillan GM, Bowman BA (2001). The prevalence of C282Y and H63D mutations in the hemochromatosis (HFE) gene in the United States. JAMA.

[25] High-resolution genotyping of HLA-DQA1 exon 1, 2 and 3 in the GoKinD Study and the identification of novel alleles DQA1*040102, *0402 and *0404. (SK Cordovado, unpublished data, Tissue Antigens 2005).

[26] Greene CN, Cordovado SK, Mueller PW (2004). Polymorphism scan for differences between transmitted and nontransmitted DRB1 *030101 alleles outside of exon 2 for type 1 diabetes: the frequency of polymorphisms is similar. Hum Immunol.

[27] Cordovado SK, Simone AE, Mueller PW (2001). High-resolution sequence-based typing strategy for HLA-DQA1 using SSP-PCR and subsequent genotyping analysis with novel spreadsheet program. Tissue Antigens.

[28] Yoon PW, Rasmussen SA, Lynberg MC, Moore CA, Anderka M, Carmichael SL (2001). The National Birth Defects Prevention Study. Public Health Rep.

[29] Rose G (1985). Sick individuals and sick populations. Int J Epidemiol.

[30] Rockhill B (2005). Theorizing about causes at the individual level while estimating effects at the population level: implications for prevention. Epidemiology.

[31] Khoury MJ, Yang Q, Gwinn M, Little J, Dana Flanders (2004). An epidemiologic assessment of genomic profiling for measuring susceptibility to common diseases and targeting interventions. Genet Med.

[32] Wacholder S (2005). The impact of a prevention effort on the community. Epidemiology.

[33] Vineis P, Christiani DC (2004). Genetic testing for sale. Epidemiology.

[34] Centers for Disease Control and Prevention (2004). Genetic testing for breast and ovarian cancer susceptibility: evaluating direct-to-consumer marketing --- Atlanta, Denver, Raleigh-Durham, and Seattle, 2003. MMWR.

[35] Centers for Disease Control and Prevention Model System for collecting, analyzing and disseminating information on genetic tests.

[36] Burke W, Thomson E, Khoury MJ, McDonnell SM, Press N, Adams PC (1998). Hereditary hemochromatosis: gene discovery and its implications for population-based screening. JAMA.

[37] Reyes M, Dunet D, Blanck HM, Grossniklaus D Hemochromatosis: information and resources for health care providers.. Genomics and population health, United States 2003.

[38] Association of State and Territorial Health Officials Genomics: a guide for public health.

[39] University of Michigan Center for Genomics and Public Health Six weeks to genomics awareness (Webcast).

[40] Partnership for Prevention Harnessing genetics to prevent disease and improve health: a state policy guide 2003.

[41] Institute of Medicine (2003). Who will keep the public healthy? Educating public health professionals for the 21st century.

[42] Shortell SM, Weist EM, Sow MS, Foster A, Tahir R (2004). Implementing the Institute of Medicine's recommended curriculum content in schools of public health: a baseline assessment. Am J Public Health.

[43] Centers for Disease Control and Prevention Genomics competencies for the public health workforce.

[44] Centers for Disease Control and Prevention Centers for Genomics and Public Health [homepage on the Internet].

[45] Sing J, Hutsell CA State capacity grants for integrating genomics into chronic disease prevention programs. In: Genomics and population health, United States 2003.

[46] Roper W Presentation at the Centers for Disease Control and Prevention's Public Health Genomics Symposium.

